# Radiation recall pneumonitis induced by chemotherapy after thoracic radiotherapy for lung cancer

**DOI:** 10.1186/1748-717X-6-24

**Published:** 2011-03-06

**Authors:** Xiao Ding, Wei Ji, Junling Li, Xiangru Zhang, Luhua Wang

**Affiliations:** 1 Department of Radiation Oncology, Cancer Institute (Hospital), Chinese Academy of Medical Sciences and Peking Union Medical College, Beijing, PR China; 2 Department of Medical Oncology, Cancer Institute (Hospital), Chinese Academy of Medical Sciences and Peking Union Medical College, Beijing, PR China; 3 Department of Radiation Oncology, Zhong Shan Hospital, Fudan University, Shanghai, PR China

## Abstract

**Background:**

Radiation recall pneumonitis (RRP) describes a rare reaction in previously irradiated area of pulmonary tissue after application of triggering agents. RRP remains loosely characterized and poorly understood since it has so far only been depicted in 8 cases in the literature. The objective of the study is to disclose the general characteristics of RRP induced by chemotherapy after thoracic irradiation for lung cancer, and to draw attention to the potential toxicity even after a long time interval from the previous irradiation.

**Methods:**

Medical records were reviewed. RRP induced by chemotherapy was diagnosed by the history of chemotherapy after radiotherapy, clinical presentation and radiographic abnormalities including ground-glass opacity, attenuation, or consolidation changes within the radiation field, plus that radiographic examination of the thorax before showed no radiation pneumonitis. RRP was graded according to Common Terminology Criteria for Adverse Events version 3.0. The characteristics of the 12 RRP cases were analyzed.

**Results:**

Twelve patients were diagnosed of RRP, of who 8 received taxanes. The median time interval between end of radiotherapy and RRP, between end of radiotherapy and beginning of chemotherapy, and between beginning of chemotherapy and RRP was 95 days, 42 days and 47 days, respectively. Marked symptomatic and radiographic improvement was observed in the 12 patients after withdrawal of chemotherapy and application of systemic corticosteroids. Seven patients were rechallenged with chemotherapy, of whom four with the same kind of agents, and showed no recurrence with steroid cover.

**Conclusions:**

Doctors should pay attention to RRP even after a long time from the previous radiotherapy or after several cycles of consolidation chemotherapy. Taxanes are likely to be associated with radiation recall more frequently. Withdrawal of causative agent and application of steroids are the treatment of choice. Patients may be rechallenged safely with steroid cover and careful observation, which needs to be validated.

## Background

Radiation recall reaction (RRR) refers to an inflammatory reaction within the previously treated radiation field in response to precipitating agents, which could have been masked if radiotherapy is not followed by inciting agents. It has been observed mainly with chemotherapeutic drugs [1]. Nevertheless, antituberculosis drugs, antibiotics, tamoxifen, simvastatin have also been involved in it [2-6]. Skin is the major site of radiation recall toxicity [7]. But it has been as well described in different internal organs including lung, digestive tract, muscle, central nervous system, and supraglottis [8-16]. Treatment-related pneumonitis is a major dose-limiting toxicities resulting from thoracic radiotherapy and chemotherapy. Radiation recall pneumonitis (RRP) describes a rare reaction in previously irradiated area of pulmonary tissue after application of triggering agents. The diagnosis of RRP induced by chemotherapy is established by a history of chemotherapy after thoracic radiotherapy, radiographic abnormality, and clinical presentation. The typical radiologic changes of RRP include ground-glass opacity, diffuse haziness, infiltrates or consolidation in the irradiated lung that conform to the shape and size of the treatment portals [17]. The symptoms are dry cough, low-grade fever, chest pain, and shortness of breath. The antineoplastic agents having been reported to trigger RRP include taxanes, anthracyclines, gemcitabine and erlotinib [8,18-23].

RRP remains loosely characterized and poorly understood since it has so far only been depicted in 8 cases [8,18-23] in the literature. The objective of the present study is to disclose the general characteristics of RRP induced by chemotherapy after thoracic irradiation of lung cancer, and to draw attention to the potential toxicity even after a long time interval from the previous irradiation.

## Methods

We retrospectively reviewed the medical and radiation records of lung cancer patients who were treated consecutively between January 1999 and December 2007 in the Department of Radiation Oncology at Cancer Hospital, Chinese Academy of Medical Sciences, Peking Union Medical College. Patients were included if they had newly diagnosed and pathologically confirmed lung cancer, chemotherapy after thoracic radiotherapy, a lung dose-volume histogram (DVH) that was recoverable from institutional archives, and availability of both radiographic images and symptom assessment for determining the occurrence of RRP.

The total normal lung volume was defined as the total lung volume minus the primary gross target volume (GTV) and volume of the trachea and main bronchi. The following dosimetric parameters were generated from the DVH for total normal lung: mean lung dose (MLD), and lung volumes receiving more than 5 Gy (V5), 10 Gy (V10), 20 Gy (V20), and 30 Gy (V30).

All patients were examined by their treating radiation oncologists weekly during radiotherapy and 4-6 weeks after completion of radiotherapy. The patients were then followed every 3 months for the first 3 years and every 6 months thereafter unless they had symptoms that required immediate examination or intervention. Radiographic examination by chest X-ray or CT was performed at each follow-up visit after completion of radiotherapy.

RRP induced by chemotherapy was diagnosed by the history of chemotherapy after radiotherapy, clinical presentation and radiographic abnormalities including ground-glass opacity, attenuation, or consolidation changes within the radiation field, plus that radiographic examination of the thorax before showed no radiation pneumonitis. RRP was graded according to the National Cancer Institute's Common Terminology Criteria for Adverse Events (CTC) version 3.0 (23) as follows: Grade 1 pneumonitis was asymptomatic and diagnosed by radiographic findings only; Grade 2 pneumonitis was symptomatic but did not interfere with daily activities; Grade 3 pneumonitis was symptomatic and interfered with daily activities or required administration of oxygen to the patient; Grade 4 pneumonitis required assisted ventilation for the patient; and Grade 5 pneumonitis was fatal. Informed consent was obtained from all the subjects.

## Results

Twelve patients were diagnosed of RRP induced by consolidation chemotherapy. The median age of the group was 51 years (range, 41-66 years). 5 patients were female, and 7 male. Three cases are limited small cell lung cancer (SCLC), and 9 are locally-advanced non small cell lung cancer (NSCLC). All patients' Karnofsky performance status (KPS) was 80. Five patients had induction chemotherapy, and 7 had concurrent chemotherapy. The 12 lung cancer patients' clinical characteristics are shown in Table 1.

**Table 1:** Clinical characteristics of the 12 lung cancer patients

Patient	Sex	Age	Histology	**Stage**^**a**^	KPS	Induction chemotherapy	Concurrent chemotherapy
1	F	51	small cell lung cancer	IIIa T1N2M0	80	CE	No
2	F	50	Adenocarcinoma	IIIb T2N3M0	80	No	PC
3	M	54	Squamous cell carcinoma	IIIa T3N2M0	80	No	EP
4	M	48	small cell lung cancer	IIIa T2N2M0	80	CE	No
5	F	59	Adenocarcinoma	IIIb T3N3M0	80	NP	No
6	M	49	Squamous cell carcinoma	IIIa T3N2M0	80	PC	EP
7	M	58	Squamous cell carcinoma	IIIa T2N2M0	80	No	EP
8	F	63	Adenocarcinoma	IIIa T2N2M0	80	No	PC
9	M	44	Squamous cell carcinoma	IIIb T4N0M0	80	No	PC
10	F	41	Adenocarcinoma	IIIb T4N2M0	80	No	EP
11	M	46	small cell lung cancer	IIIa T2N2M0	80	EP	No
12	M	66	Squamous cell carcinoma	IIIa T2N2M0	80	No	No

KPS indicates Karnofsky performance status; CE, carboplatin, etoposide; NP, navelbine, cisplatin; PC, Paclitaxel, carboplatin; EP, etoposide, cisplatin.

^a ^Grading determined according to the American Joint Committee on Cancer 6th edition grading system.

Eight patients received 3-dimentional conformal radiotherapy (3D-CRT), and 4 received intensity-modulated radiotherapy (IMRT). The median radiation dose was 60.7 Gy (range, 52-66 Gy). The median MLD was 1540.5 cGy (range, 1301-2130 cGy). The median V5, V10, V20 and V30 was 53.3% (range, 38.0%-65.0%), 41.0% (range, 29.0%-51.0%), 26.9% (range, 20.0%-32.0%), and 20.2% (range, 15.0%-27.0%), respectively. The 12 lung cancer patients' dosimetric parameters are shown in Table 2.

**Table 2:** Dosimetric parameters of the 12 lung cancer patients

Patient	Radiotherapy	MLD(cGy)	V5(%)	V10(%)	V20(%)	V30(%)
1	3D-CRT 60Gy/30F/41D	1560	46.0	41.0	26.5	22.0
2	IMRT 54Gy/24F/37D	1489	63.0	47.0	27.0	19.0
3	3D-CRT 62.6Gy/34F/36D	1591	49.0	40.0	29.0	22.0
4	IMRT 60Gy/30F/39D	1319	55.0	38.0	24.0	15.0
5	3D-CRT 52Gy/26F/36D	1819	65.0	51.0	32.0	27.0
6	3D-CRT 63Gy/35F/56D	2130	62.0	44.0	28.0	21.0
7	3D-CRT 61.4Gy/34F/48D	1301	42.0	30.0	20.0	17.0
8	3D-CRT 63Gy/35F/52D	1521	39.9	33.5	24.7	20.5
9	3D-CRT 64.6Gy/35F/53D	1755	38.0	29.0	22.0	19.0
10	IMRT 66Gy/33F/45D	1667	57.2	40.9	27.8	19.9
11	IMRT 60Gy/30F/38D	1444	54.5	43.7	26.8	19.0
12	3D-CRT 56Gy/28F/38D	1445	52.0	46.0	28.0	22.0

MLD indicates mean lung dose; 3D-CRT, 3-dimensional conformal radiotherapy; IMRT, intensity-modulated radiotherapy.

Of the 12 intravenous consolidation chemotherapy regimens inducing RRP, 8 included taxanes, 2 of which included both taxanes and gemcitabine; 2 etoposide; 1 vinorelbine; and 1 epirubicin.

The median time interval between end of radiotherapy and RRP, between end of radiotherapy and beginning of chemotherapy, and between beginning of chemotherapy and RRP was 95 days (range, 71-202 days), 42 days (range, 7-60 days) and 47 days (range, 22-169 days), respectively.

Eleven patients had Grade 2 and 1 patient had Grade 3 RRP. Marked symptomatic and radiographic improvement was observed in the 12 patients after withdrawal of the chemotherapy and application of systemic corticosteroids. Of the 12 RRP patients, 7 were rechallenged with chemotherapy, 3 of who were rechallenged with the same agents and 1 with the same kind of agents, and showed no recurrence with steroid cover. The median time interval between RRP and rechallenge was 20 days (range, 4-89 days). The characteristics of the 12 RRP cases are shown in Table 3.

**Table 3:** Characteristics of the 12 RRP cases

Patient	Consolidation chemotherapy	Time interval between end of RT and RRP (days)	Time interval between end of RT and beginning of ChT (days)	Time interval between beginning of ChT and RRP (days)	Fever	Cough Grade	Grade of shortness of breath	RRP Grade	Rechallenge	Time interval between RRP and rechallenge (days)
1	CEV×2	71	42	29	<38°C	2	2	2	No	
2	D×1	82	31	51	<38°C	3	0	3	No	
3	GD×1	81	59	22	<38°C	0	0	2	GD	14
4	CE×4	94	15	79	No	2	0	2	P	89
5	NP×2	102	60	42	<38°C	0	2	2	No	
6	PC×2	86	46	40	<38.5°C	2	2	2	D	85
7	GD×2	94	56	38	<38°C	0	0	2	GD	20
8	PC×1	95	59	36	<38°C	2	0	2	PC	20
9	PC×2	105	34	71	No	2	2	2	NP	73
10	PC×2	118	41	77	<38°C	1	2	2	No	
11	EP×4	171	7	164	No	2	0	2	GI	4
12	PC×3	202	31	169	<38°C	2	0	2	No	

RRP indicates radiation recall pneumonitis; CEV, cyclophosphamide, epirubicin, vincristine; D, docetaxel; GD, gemcitabine, docetaxel; CE, carboplatin, etoposide; NP, navelbine, cisplatin; PC, paclitaxel, carboplatin; EP, etoposide, cisplatin; RT, radiotherapy; ChT, chemotherapy; P, paclitaxel; GI, gemcitabine, ifosfamide.

**Figure 1:**
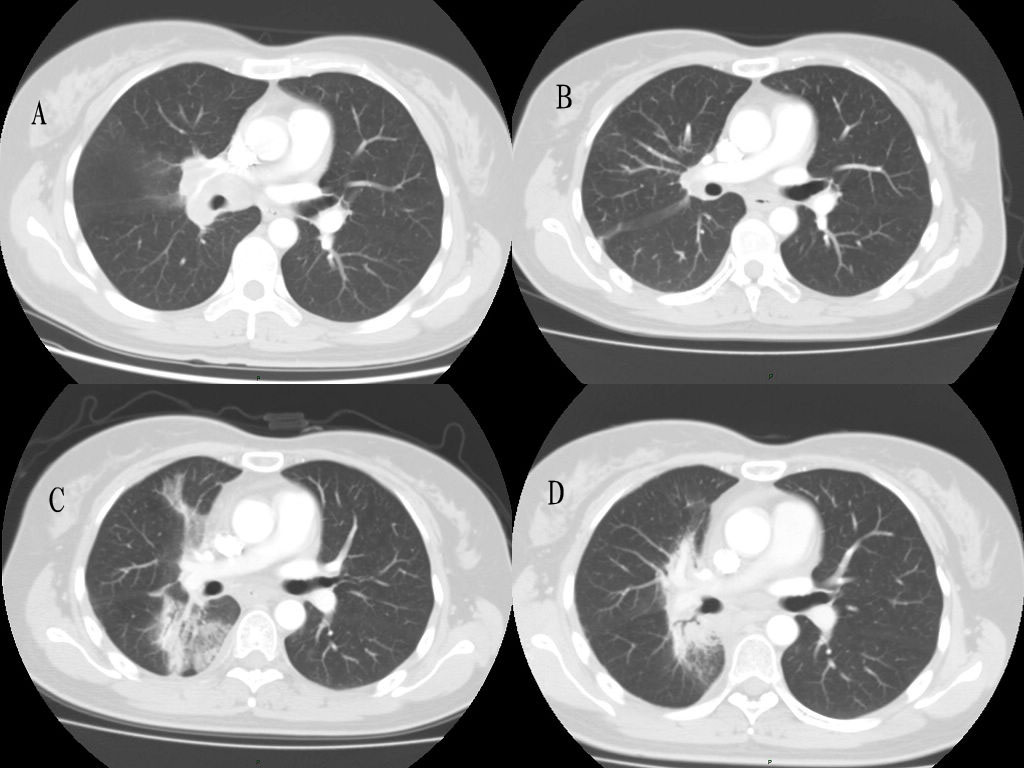
**Thoracic CT scans of Patient 10 (A) before radiotherapy, (B) one month after end of radiotherapy, (C) 4 months after end of radiotherapy when RRP took place induced by consolidation chemotherapy, and (D) days after application of systematic steroids**. (A)(B): No pulmonary infiltrate, (C): Pulmonary ground-glass opacity, (D): Partial resolution of the lung infiltrate.

**Figure 2:**
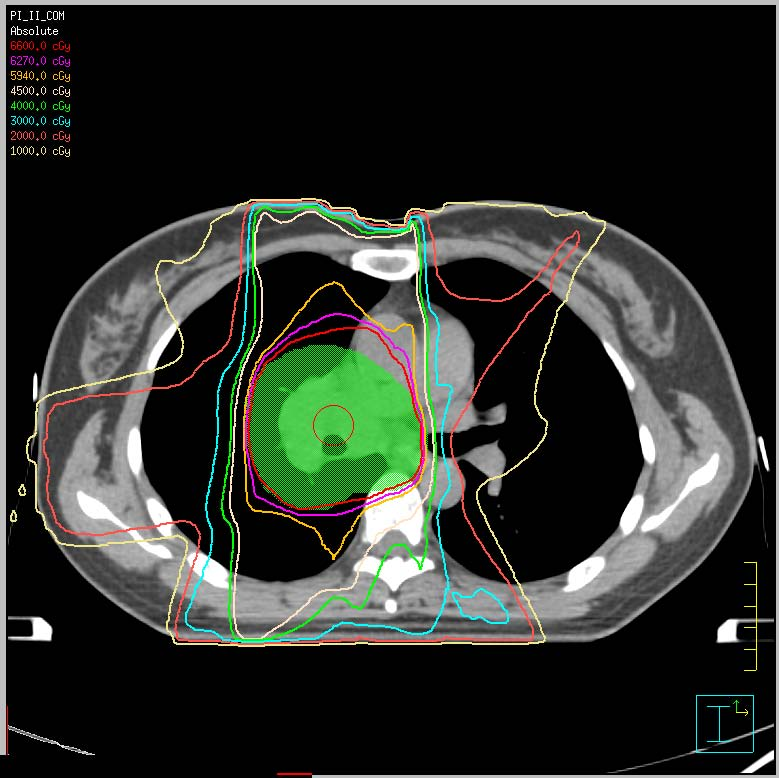
**CT based IMRT plan of Patient 10**.

Figure 1 shows the thoracic CT scans of Patient 10 (A) before radiotherapy, (B) one month after end of radiotherapy, (C) 4 months after end of radiotherapy when RRP took place induced by consolidation chemotherapy, and (D) days after application of systematic steroids, suggestive of RRP development. Figure 2 shows CT based IMRT plan of Patient 10.

## Discussion

RRR describes an inflammatory reaction in previously irradiated area after application of certain promoting agents. When it occurs in previously irradiated lung, it is called RRP. RRP is a special subtype of radiation induced pneumonitis, as the base of RRP is subclinical radiation damage of pulmonary tissue. When radiation therapy is followed by chemotherapy, subclinical damage from irradiation can be unmasked and clinically manifested as a radiation recall phenomenon.

Taxanes and anthracyclines have been reported to be responsible for 20% and nearly 30% of RRR, respectively [1]. The inciting agents observed in RRP previously reported and here included taxanes, anthracyclines, gemcitabine, etoposide, vinorelbine and erlotinib. Taxanes and anthracyclines are responsible for the majority of the 20 chemotherapy-induced RRP cases available, 50% and 25% respectively. In the present study, of the 12 regimens, 8 (66.7%) included taxanes, 2 (16.7%) of which included both taxanes and gemcitabine; 2 (16.7%) etoposide; 1 vinorelbine; and 1 epirubicin. Certain drugs seem to be associated with radiation recall more frequently. On the other hand, cisplatin and carboplatin, which are frequently used after radiotherapy, has not been depicted in RRR. In contrast, radiation recall induced by oxaliplatin has been reported [24]. When a combination of gemcitabine and docetaxel was involved, we assume that RRP was induced by the combination, as it could not completely be ruled out that the pulmonary recall reaction was not caused by either, although the time intervals from the last application of the two agents to the RRP were different.

So far, we are the first to describe etoposide-induced RRP with details. Moreover, we are the first to describe RRR by Vinorelbine beyond one suspected RRR case after a first cycle of gemcitabine and Vinorelbine with no details [22].

Classic RRR often occurs with the initiation of the precipitating agent but can occur after several courses of treatment. The time delay of cases that occurred after several courses of treatment could be explained by a putative drug dose threshold for RRP or/and a time lag effect. Clinically, these patients' symptoms were considered to be triggered by chemotherapy. Both radiotherapy and chemotherapy contributed to the development of RRP, and it is difficult to tell how much each of them contributed in each case. The reported time interval between the end of radiation therapy and the recall reaction ranged from 2 days [25] to 15 years [26]. The reported time interval between the first dose of chemotherapy and the recall reaction ranged from 18 hours [27] to 15 years [26]. In the literature, the time interval between completion of radiotherapy and RRP ranged from 12 days [8] to 9 months [21], the time interval between completion of radiotherapy and beginning of chemotherapy ranged from 12 days [8] to 8 months [21], and the time interval between beginning of chemotherapy and RRP ranged from several hours [8] to 2 months [19]. In the present study, the median time interval between end of radiotherapy and RRP, between end of radiotherapy and beginning of chemotherapy, and between beginning of chemotherapy and RRP was 95 days (range, 71-202 days), 42 days (range, 7-60 days) and 47 days (range, 22-169 days), respectively. RRP could occur even after a long time interval from the previous radiotherapy or after several cycles of consolidation chemotherapy. Because we generally recommend our patients have consolidation chemotherapy 4-8 weeks after radiotherapy in our institute if the patients are evaluated able to take chemotherapy. The time interval from the end of radiotherapy to RRP here could not be very long.

Previous published articles have reported that recall reactions are most severe when the time interval between the radiotherapy and the following chemotherapy is short. We did not find the trend in our study, the reason for that may be there are other factors, such as primary disease, patient's performance status, radiotherapy and inciting agents. Referring to all the chemotherapy-induced RRP cases reported and here, the median time interval from completion of radiotherapy to beginning of chemotherapy was 34 days (range, 12-59 days) for taxanes, 6 weeks (range, 3-8 weeks) for anthracyclines, 59 days (range, 56 days-8 months) for combination of gemcitabine and docetaxel; the median time interval from beginning of chemotherapy and RRP was 51 days (range, 36 hours-169 days) for taxanes, 12 hours (range, several hours-2 months) for anthracyclines, 30 days (range, 22-38 days) for combination of gemcitabine and docetaxel; and the median time interval from completion of radiotherapy and RRP was 95 days (range, 12-202 days) for taxanes, 6 weeks (range, 3 weeks-4 months) for anthracyclines, 94 days (range, 81 days-9 months) for combination of gemcitabine and docetaxel. Probably, the time interval plays a crucial role in the pathophysiological mechanism.

Standard treatment for radiation recall includes withdrawal of the precipitating agent, application of corticosteroids and supportive care. Marked symptomatic and radiographic improvement has been observed in all the 12 patients after withdrawal of the chemotherapy and application of systemic corticosteroids. The most confusing aspect in the treatment of RRP is to decide whether to give up the inciting drug even chemotherapy or not. This must be considered since it means that an effective treatment of a patient's malignancy stops. Of our 12 RRP patients, 7 were rechallenged with chemotherapy, of which 3 were rechallenged with the same agents and 1 with the same kind of agents, and showed no recurrence with steroid cover. In the literature 2 RRP patients rechallenged with adriamycin [19] and paclitaxel [8] respectively showed no recurrence with steroid cover. As for radiation recall dermatitis (RRD) that has relatively more evidence of rechallenge in the literature, drug rechallenge tends to produce either only a mild recurrence or no recurrence of recall [7]. Hence, it may work to rechallenge RRP patient with the same agent with steroid cover and careful observation, which needs more data to verify. However, it should be noted that our 12 patients whose KPS was 80 received 3D-CRT or IMRT. Furthermore, with steroid cover, we only rechallengd the patients whom we clinically assessed could take it. Also, it is possible that the rechallenged patients may have showed recurrence without steroid cover or selection.

The etiology and pathogenesis of RRR are not completely understood. One hypothesis is that local vascular permeability or proliferative changes induced by radiotherapy might affect the subsequent pharmacokinetics of the inciting drug [28]. Another is that after radiotherapy permanent changes had been induced in stem cells' functional features, such as capacity of proliferation, consequently the reaction occurs when the stem cells are exposed to a triggering agent [29]. Nevertheless, Abadir and Liebmann [30] suggest that the stem cells cycle at a faster rate to maintain an adequate functioning on the irradiated zone, thus they are more susceptible to be damaged by active drugs. However, the absence of recurrence in cases that were rechallenged with the same drug, and reactions caused by noncytotoxic drugs do not support these hypotheses. Camidge and Price [19] reported that the role of idiosyncratic drug reactions should be emphasized more than the cytotoxicity of the drug due to the rarity of reaction, the speed of onset, and the extreme drug specificity. They also reported that radiation recall dermatitis may represent the koebner phenomenon [31]. No recurrence each time after rechallenge with the same drug supports the theory of drug hypersensitivity reaction. Further studies are needed to elucidate the etiology and pathogenesis of RRR.

## Conclusions

Although RRP is a rarely reported phenomenon after previous thoracic radiotherapy, doctors should pay attention to this potential toxicity even after a long time interval from the previous radiotherapy or after several cycles of consolidation chemotherapy. Withdrawal of the causative agent and application of systematic steroids are the treatment of choice. Patients may be rechallenged safely with the same agent with steroid cover and careful observation, which needs more data to verify.

## Competing interests

The authors declare that they have no competing interests.

## Authors' contributions

JL and XZ participated in the design and coordination of the study, and helped to analyze the data. LW, XD, and WJ conceived of the study, and participated in its design and coordination, and helped to analyze the data and draft the manuscript. All authors read and approved the final manuscript.

## References

[B1] Azria D Magné N Zouhair A Castadot P Culine S Ychou M Stupp R Van Houtte P Dubois JB Ozsahin M Radiation recall: a well recognized but neglected phenomenon Cancer Treat Rev 2005 31 555 570 10.1016/j.ctrv.2005.07.008 16168567

[B2] Extermann M Vogt N Forni M Dayer P Radiation recall in a patient with breast cancer treated for tuberculosis Eur J Clin Pharmacol 1995 48 77 78 10.1007/BF00202177 7621852

[B3] Garza LA Yoo EK Junkins-hopkins JM VanVoorhees AS Photo recall effect in association with cefazolin Cutis 2004 73 79 85 14964636

[B4] Parry BR Radiation recall induced by tamoxifen Lancet 1992 340 49 10.1016/0140-6736(92)92460-W 1351618

[B5] Singer EA Warren RD Pennanen MF Collins BT Hayes DF Tamoxifen-induced radiation recall dermatitis Breast J 2004 10 170 171 10.1111/j.1075-122X.2004.21222.x 15009054

[B6] Abadir R Liebmann J Radiation reaction recall following simvastatin therapy: a new observation Clin Oncol 1995 7 325 326 10.1016/S0936-6555(05)80545-X 8580062

[B7] Camidge R Price A Characterizing the phenomenon of radiation recall dermatitis Radiother Oncol 2001 59 237 245 10.1016/S0167-8140(01)00328-0 11369064

[B8] Schweitzer VG Juillard GJ Bajada CL Parker RG Radiation recall dermatitis and pneumonitis in a patient treated with paclitaxel Cancer 1995 76 1069 1072 10.1002/1097-0142(19950915)76:6<1069::AID-CNCR2820760623>3.0.CO;2-7 8625210

[B9] Jeter MD Pasi AJ Brooks S Burstein HJ Wen P Fuchs CS Loeffler JS Devlin PM Salgia R Gemcitabine-induced radiation recall Int J Radiat Oncol Biol Phys 2002 53 394 400 10.1016/S0360-3016(02)02773-6 12023144

[B10] Showel J Hoover SV Deutsch S Radiation-recall Int J Radiat Oncol Biol Phys 1993 25 929 10.1016/0360-3016(93)90330-X 8478249

[B11] Stein RS Radiation-recall enteritis after actinomycin-D and adriamycin therapy South Med J 1978 71 960 961 684478 10.1097/00007611-197808000-00024

[B12] Kundak I Oztop I Soyturk M Ozcan MA Yilmaz U Meydan N Gorken IB Kupelioglu A Alakavuklar M Paclitaxel-carboplatin induced radiation recall colitis Tumori 2004 90 256 258 15237594 10.1177/030089160409000219

[B13] Friedlander PA Bansal R Schwartz L Wagman R Posner J Kemeny N Gemcitabine-related radiation recall preferentially involves internal tissue and organs Cancer 2004 100 1793 1799 10.1002/cncr.20229 15112258

[B14] Ganem G Solal-Celigny P Joffroy A Tassy D Delpon A Dupuis O Radiation myositis: the possible role of gemcitabine Ann Oncol 2000 11 1615 1616 10.1023/A:1008353224251 11205473

[B15] Wallenborn PA Postma DS Radiation recall supraglottitis. A hazard in head and neck chemotherapy Arch Otolaryngol 1984 110 614 617 6477283 10.1001/archotol.1984.00800350056015

[B16] Wiatrak BJ Myer CM Radiation recall supraglottitis in a child Am J Otolaryngo 1991 12 227 229 10.1016/0196-0709(91)90122-V 1767873

[B17] Choi YW Munden RF Erasmus JJ Park KJ Chung WK Jeon SC Park CK Effects of radiation therapy on the lung: Radiologic appearances and differential diagnosis Radiographics 2004 24 985 998 10.1148/rg.244035160 15256622

[B18] Ma LD Taylor GA Wharam MD Wiley JM ''Recall'' pneumonitis: adriamycin potentiation of radiation pneumonitis in two children Radiology 1993 187 465 467 8475291 10.1148/radiology.187.2.8475291

[B19] McLnerney DP Bullimore J Reactivation of radiation pneumonitis by adriamycin Br J Radiol 1977 50 224 227 10.1259/0007-1285-50-591-224 191139

[B20] Hill AB Tattersall SF Recall of radiation pneumonitis after intrapleural administration of doxorubicin Med J Aust 1983 1 39 40 6571523 10.5694/j.1326-5377.1983.tb136023.x

[B21] Schwarte S Wagner K Karstens JH Bremer M Radiation recall pneumonitis induced by gemcitabine Strahlenther Onkol 2007 183 215 217 10.1007/s00066-007-1688-z 17406804

[B22] Castellano D Hitt R Ciruelos E Cortés-Funes H Colomer R Biweekly vinorelbine and gemcitabine: a phase I dose-finding study in patients with advanced solid tumors Ann Oncol 2003 14 783 787 10.1093/annonc/mdg196 12702534

[B23] Togashi Y Masago K Mishima M Fukudo M Inui K A case of radiation recall pneumonitis induced by erlotinib, which can be related to high plasma concentration J Thorac Oncol 2010 5 924 925 10.1097/JTO.0b013e3181dab0dd 20502274

[B24] Chan RT Au GK Ho JW Chu KW Radiation recall with oxaliplatin: report of a case and a review of the literature Clin Oncol 2001 13 55 57 10.1053/clon.2001.9216 11292139

[B25] Raghavan VT Bloomer WD Merkel DE Taxol and radiation recall dermatitis Lancet 1993 341 1354 10.1016/0140-6736(93)90871-D 8098490

[B26] Burdon J Bell R Sullivan J Henderson M Adriamycin-induced recall phenomenon 15 years after radiotherapy Jama 1978 239 931 10.1001/jama.239.10.931b 628037

[B27] Kellie SJ Plowman PN Malpas JS Radiation recall and radiosensitization with alkylating agents Lancet 1987 1 1149 1150 10.1016/S0140-6736(87)91711-9 2883478

[B28] Bostrom A Sjolin-Forsberg G Wilking N Bergh J Radiation recall - another call with tamoxifen Acta Oncol 1999 38 955 959 10.1080/028418699432653 10606426

[B29] Seymour CB Mothersill C Alper T High yields of lethal mutations in somatic mammalian cells that survive ionizing radiation Int J Radiat Biol Relat Stud Phys Chem Med 1986 50 167 179 10.1080/09553008614550541 3487520

[B30] Abadir R Liebmann J Radiation reaction recall following simvastatin therapy: a new observation Clin Oncol (R Coll Radiol) 1995 7 325 326 8580062 10.1016/s0936-6555(05)80545-x

[B31] Camidge R Price A Radiation recall dermatitis may represent the koebner phenomenon J Clin Oncol 2002 20 4130 10.1200/JCO.2002.99.148 12351618

